# Effect of calcium cyanamide, ammonium bicarbonate and lime mixture, and ammonia water on survival of *Ralstonia solanacearum* and microbial community

**DOI:** 10.1038/srep19037

**Published:** 2016-01-07

**Authors:** Lijuan Liu, Chengliang Sun, Xingxing Liu, Xiaolin He, Miao Liu, Hao Wu, Caixian Tang, Chongwei Jin, Yongsong Zhang

**Affiliations:** 1 Ministry of Education Key Laboratory of Environmental Remediation and Ecosystem Health, College of Environmental and Resource Sciences, Zhejiang University, Hangzhou 310058, China; 2 Zhejiang Provincial Key Laboratory of Subtropical Soil and Plant Nutrition, College of Environmental and Resource Sciences, Zhejiang University, Hangzhou 310058, China; 3 Centre for AgriBioscience/Department of Agricultural Sciences, La Trobe University, Melbourne Campus, Bundoora, Vic 3086, Australia

## Abstract

The inorganic nitrogenous amendments calcium cyanamide (CC), ammonia water (AW), and a mixture of ammonium bicarbonate with lime (A+L) are popularly used as fumigants to control soil-borne disease in China. However, it is unclear which of these fumigants is more effective in controlling *R. solanacearum*. This present study compared the efficiencies of the three nitrogenous amendments listed above at four nitrogen levels in suppressing the survival of *R. solanacearum* in soil. The CC showed the best ability to suppress *R. solanacearum* due to its highest capacity to increase soil 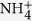 and NO_2_^−^ contents and pH. However, AW was more suitable to controlling bacterial wilt caused by *R. solanacearum* because it had a lower cost and its application rate of 0.25 g N kg^−1^ soil could effectively suppress the survival of *R. solanacearum*. Additionally, soil microbial activity and community populations were restored to their initial state four weeks after the application of each fumigant, indicating that the three fumigants had few detrimental impacts on soil microbial activity and community structure with an exception of the suppression of *R. solanacearum*. The present study provides guidance for the selection of a suitable alkaline nitrogenous amendment and its application rate in controlling bacterial wilt.

Bacterial wilt is a systemic vascular disease in plants caused by *Ralstonia solanacearum*, an important soil-borne bacterial pathogen^1^. This pathogen can survive in soils and water bodies for an extended duration until it enters host plants via the roots^2^. Once the population of *R. solanacearum* exceeds 10^6^ CFU g^−1^ dry soil, wilt is difficult to control and results in severe yield losses of many economically relevant crops such as potato, tomato, tobacco, pepper, eggplant, banana, ginger and geranium^3^,^4^,^5^,^6^.

Many strategies have been proposed to control bacterial wilt. These include planting resistant varieties, transgenic-resistant plants and crop rotation. However, limited success has been achieved due to the high survival capacity of *R. solanacearum* in complex environments, a wide host range, and broad geographic distribution and genetic diversity^5^,^7^,^8^. Soil pre-treatment has recently been widely used to control soil-borne pathogens, such as soil fumigation with methyl bromide, methyl iodide, and propargyl bromide, and these methods have achieved good outcomes^9^. However, the use of fumigant pesticides has been restricted in many countries due to their environmental risks^10^,^11^. Some physical methods, such as solarization, steaming and heating, have also been employed to control soil-borne disease. However, the efficiency of physical methods is limited by climatic conditions, soil type and the soil water content^12^,^13^. Therefore, new effective alternative strategies with fewer environmental risks are needed to control soil-borne *R. solanacearum*.

Previous studies showed that inorganic or organic nitrogenous amendments could suppress the survival of soil-borne pathogens through the accumulation of different nitrogen forms (NH_3_, 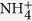, NO_2_^−^, and NO_3_^−^) in soil^14^,^15^,^16^. Additionally, many studies have also shown that the control efficiency of nitrogenous materials against pathogens could be improved by increasing the pH value of the soil^15^,^17^,^18^. Consequently, alkaline inorganic nitrogenous amendments have been used by growers in China to control soil-borne disease, as these amendments are locally available^18^,^19^ and also provide nitrogen sources for crops. The alkaline inorganic nitrogenous amendments commonly used in China include calcium cyanamide, a mixture of ammonium bicarbonate and lime, and ammonia water. The chemical features of these amendments are substantially different; thus, their efficiencies against soil-borne pathogens may also differ. However, little information is available on the efficiencies of these amendments. Moreover, the amount of nitrogenous materials sufficient to suppress the survival of pathogens is unknown. Therefore, it is necessary to compare the efficiency of the above three alkaline nitrogenous amendments in suppressing the survival of pathogens and to identify their optimal application rates. The suppressive capacity against soil-borne pathogens and whether the above-mentioned alkaline nitrogenous amendments affect non-target indigenous microbial communities and soil function should be investigated; potential detrimental effects on biological processes could destroy soil function and the productivity of an agricultural system.

The aims of this study were (1) to compare the effects of calcium cyanamide, ammonium bicarbonate combined with lime, and ammonia water on the survival of *R. solanacearum* in soil at four nitrogen levels in an attempt to select a nitrogenous form that has a high efficiency in suppressing pathogens, (2) to identify the optimal application rate, and (3) to monitor changes in the soil microbial community structure and function after the application of these amendments.

## Results

### Soil pH and inorganic nitrogen levels

**Figure 1:**
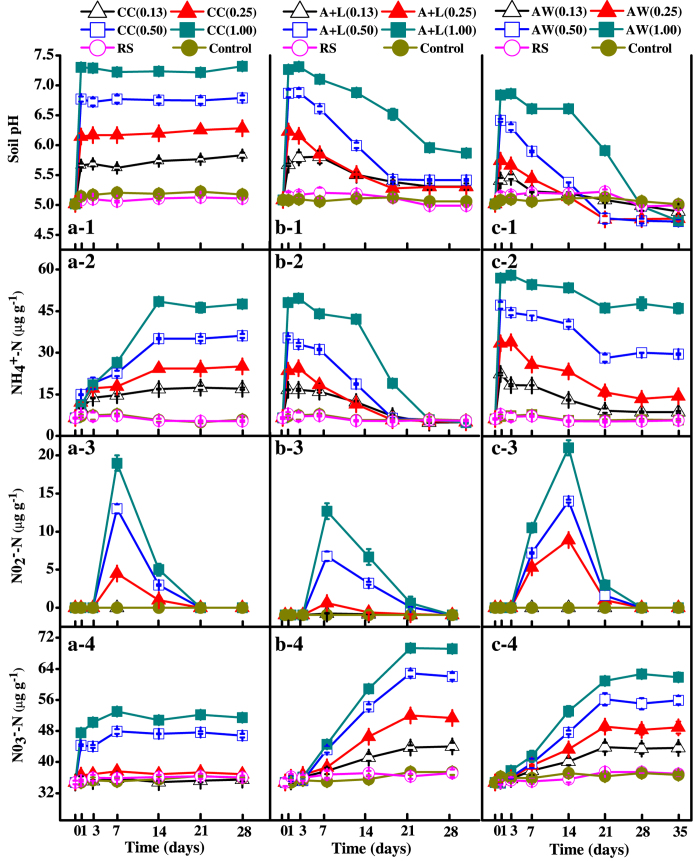
Change in soil pH (a-1–c-1), NH_4_^+^-N (a-2–c-2), NO_2_^−^-N (a-3–c-3) and NO_3_^−^-N (a-4–c-4) concentrations in calcium cyanamide (a-1–a-4), ammonium bicarbonate and lime mixture (b-1–b-4) and ammonia water (c-1–c-4) treatments. Data are the means ± SD (n = 4) shown by vertical error bars. The explanations of treatment abbreviations are referred to Table 2.

The pH and the concentrations of 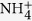-N, NO_2_^−^-N and NO_3_^−^-N in the RS and control treatments remained relatively stable during the 28-d incubation period, whereas they varied markedly in the calcium cyanamide (CC), ammonium bicarbonate and lime mixture (A+L), and ammonia water (AW) treatments (*P* < 0.05) (Fig. 1).

With increasing level of amendments, soil pH increased sharply at the initial stage and peaked within 3 days (Fig. 1a-1–c-1). The pH values ranged from 5.83 to 7.32 in the CC treatments, 5.76 to 7.35 in the A+L treatments, and 5.47 to 6.75 in the AW treatments (Fig. 1). Thereafter, the soil pH value was maintained at a significantly higher level in the CC treatments than the RS and control treatments (*P* < 0.05); however, the soil pH value gradually decreased in the A+L and AW treatments. At the end of the incubation, the A+L treatments remained at a slightly higher pH than (and the AW treatments had a similar pH to) that of the RS and control treatments (*P* < 0.05).

**Figure 2:**
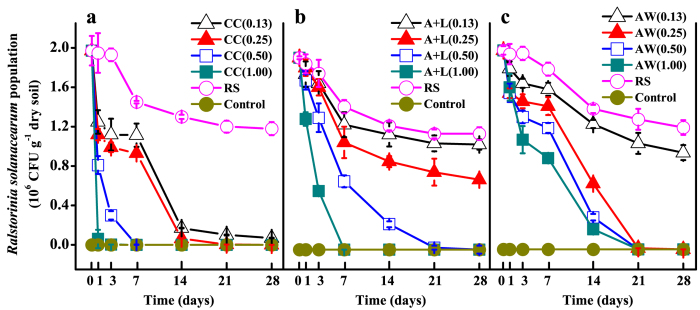
Effect of calcium cyanamide (**a**), ammonium bicarbonate and lime mixture (**b**), and ammonia water (**c**) on the survival of *Ralstonia solanacearum*. Data are the means ± SD (n = 4) shown by vertical error bars. The explanations of treatment abbreviations are referred to Table 2.

The 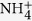-N concentration of the treated soil also showed a marked increase as the level of amendment increased (Fig. 1a-2-c-2). In the CC treatments, 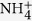-N peaked at the 14^th^ day of incubation and was then maintained at a constant level. In comparison, the 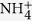-N concentration in the A+L treatments decreased sharply 1 day after the application; however, there was an initial increase at the very beginning, which decreased to nearly the same levels as those in the RS or control treatments at the end of the incubation. In the AW treatments, only slight changes were observed in the soil 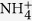-N contents throughout the incubation period after an initial increase.

**Figure 3:**
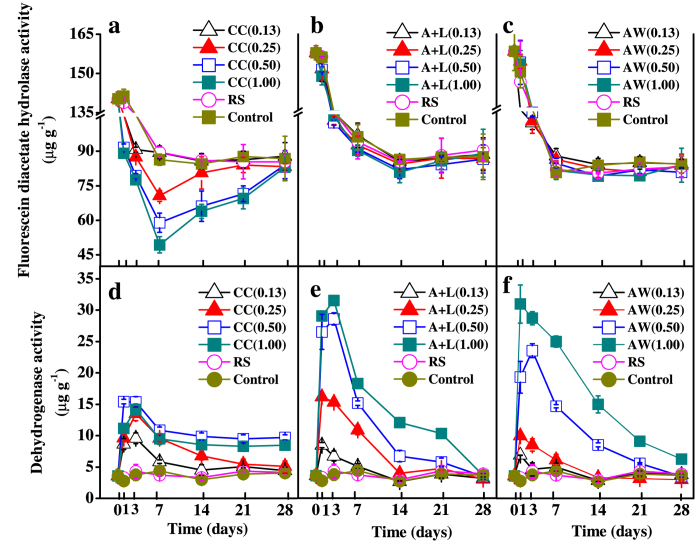
Changes in FDA hydrolysis and dehydrogenase activity after the application of calcium cyanamide (**a**,**d**), ammonium bicarbonate and lime mixture (**b**,**e**) and ammonia water (**c**,**f**), respectively. Data are the means ± SD (n = 4) shown by vertical error bars. The explanations of treatment abbreviations are referred to Table 2.

Throughout the incubation period, the soil NO_2_^−^-N was below the detection limit when the nitrogenous amendments were applied at 0.13 g N kg^−1^ soil. The soil NO_2_^−^-N increased when the N application rate was over 0.25 g N kg^−1^ soil, with the increase being significantly higher in the CC and AW treatments than in the A+L treatments (Fig. 1a-3–c-3). Soil NO_2_^−^-N content then declined sharply with time and finally decreased below the detection limit.

**Figure 4:**
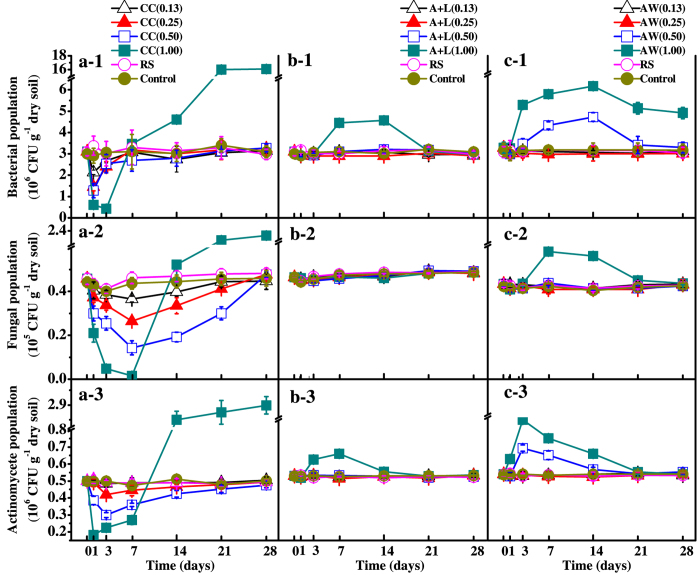
Changes of bacterial (a-1–c-1), fungal (a-2–c-2) and actinomycetes (a-3–c-3) communities populations in calcium cyanamide (a-1–a-3), ammonium bicarbonate and lime mixture (b-1–b-3) and ammonia water (c-1–c-3) treatments. Data are the means ± SD (n = 4) shown by vertical error bars. The explanations of treatment abbreviations are referred to Table 2.

Soil NO_3_^−^-N concentrations generally increased as the level of nitrogenous amendments increased (Fig. 1a-4-c-4). The NO_3_^−^-N concentrations peaked at Day 7 in the CC treatments and Day 21 in the A+L and AW treatments; the concentrations then plateaued. Unexpectedly, the soil NO_3_^−^-N concentrations were not affected by the two lower N application rates in the CC treatments. Furthermore, the NO_3_^−^-N concentrations in the CC treatments were significantly lower than those in the A+L and AW treatments at a given level of application (*P* < 0.05).

### Effects on the survival of *R. solanacearum*

As shown in Fig. 2, the *R. solanacearum* in the control soil was not detected throughout the incubation period. In the RS treatment, the populations of *R. solanacearum* declined gradually and then remained relatively stable after 7 days of inoculation. The suppression efficiency highly depends on the application rate and type of nitrogenous amendments. When the nitrogenous amendment was at a 0.13 g N kg^−1^ soil level, the calcium cyanamide substantially suppressed but the other two amendments only slightly suppressed the survival of *R. solanacearum*. At the level of 0.25 g N kg^−1^ soil, the suppression efficiency in the A+L treatment was significantly less than that of the CC and AW treatments (*P* < 0.05), in which the population of *R. solanacearum* was similar to that of the control treatment after 14 and 21 days of incubations, respectively. When the nitrogen level reached 0.50 g N kg^−1^ soil or above, the survival of *R. solanacearum* was substantially suppressed by all of the nitrogenous amendments; however, the suppression duration was shorter in the CC treatments than in the A+L or AW treatments.

### Soil fluorescein diacetate (FDA) hydrolysis and dehydrogenase activity

FDA hydrolysis is widely accepted as an accurate and simple method for measuring total microbial activity^20^. FDA hydrolysis showed a clear reducing trend within 7 days and then remained at a stable level in the control and RS treatments (Fig. 3). During the 28-d incubation period, FDA hydrolysis results in the A+L and AW treatments did not differ from those in the control and RS treatments (*P* > 0.05) (Fig. 3b,c); however, FDA hydrolysis activities decreased with the nitrogen level of the CC treatments in the first 7 days of incubation and then increased to a level similar to that in the control and RS treatments (Fig. 3a).

Dehydrogenase activity is used as a broad-spectrum indicator for the metabolic activity of microorganisms^21^. The activity of dehydrogenase in the control and RS treatments remained stable throughout the incubation period (Fig. 3) but gradually decreased after an initial increase within 3 days after commencement of CC, A+L and AW treatments (Fig. 3d–f). Specifically, at or below 0.25 g N kg^−1^ soil, the dehydrogenase activity of the CC, A+L and AW treatments was similar to that in the control and RS treatments at the end of the incubation; at a nitrogen level of 0.50 N kg^−1^ soil, dehydrogenase was significantly higher activity in the CC treatment than in the control and RS treatments after 7 days (*P* < 0.05) (Fig. 3d), whereas dehydrogenase activity was comparable among the A+L, AW, RS and control treatments (Fig. 3e,f). As the N application rate further increased (i.e., 1.00 N kg^−1^ soil), the dehydrogenase activity in the CC and AW treatments had higher values on the 3^td^ and 28^th^ day of incubation, respectively; however, dehydrogenase activity was not affected by A+L application at the end of the 28-d incubation.

### Soil microbial community

The bacterial population remained at a stable level and showed no difference between the control and nitrogenous amendments at or below 0.25 g N kg^−1^ soil during the incubation period (Fig. 4a-1–c-1). At the N level of 0.50 g kg^−1^ soil, the bacterial population was significantly decreased in the CC treatment and increased in the AW treatment during the first 7 and 21 days of incubation, respectively; following this, the bacterial population returned to a level comparable to those of the control and RS treatment. When the level of nitrogenous amendment was at 1.00 g N kg^−1^ soil, the bacteria population was greater in the CC and AW treatments; in the A+L treatment (*P* < 0.05), the population became similar to that in the control treatment after 21 days of incubation (Fig. 4b-1).

The fungal population in the CC, A+L and AW treatments at or below 0.50 g N kg^−1^ soil was similar to that in the control and RS treatments during incubation (Fig. 4a-2–c-2); however, there was a transient decrease after the application of calcium cyanamide. When the level of nitrogenous amendment was increased to 1.00 g N kg^−1^ soil, The CC treatment significantly increased the fungal population by approximately 5-fold compared with the control and RS treatments (*P* < 0.05) (Fig. 4a-2), whereas the A+L and AW did not affect the fungal population (Fig. 4b-2,c-2).

The CC, A+L and AW treatments at or below 0.25 g N kg^−1^ soil did not affect the population of actinomycetes. When nitrogenous amendments were at 0.50 g N kg^−1^ soil, the population of actinomycetes significantly decreased in the CC treatment and increased in the AW treatment during the first 3 days; the actinomycetes then recovered to their initial state. However, the actinomycete population was not affected in the A+L treatment throughout the entire 28-d incubation. At the level of 1.00 g N kg^−1^ soil, the actinomycete population in the A+L and AW treatments remained at a level similar to the control after 21 days; however, it decreased in the first 7 days but substantially increased from Day 14 in the CC treatment.

### Soil microbial community structure

**Figure 5:**
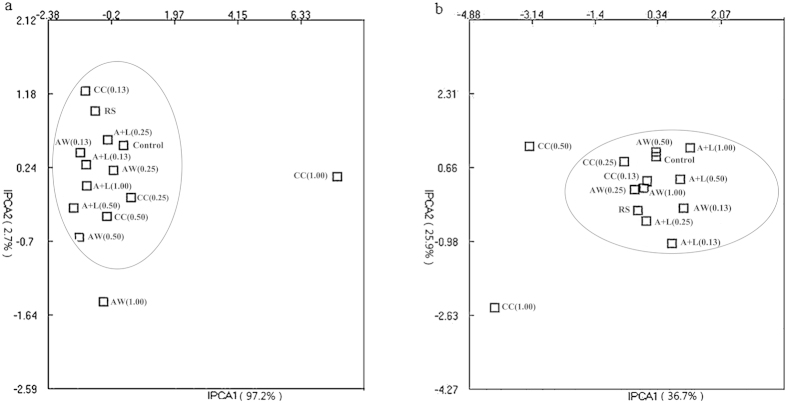
Principal component analysis of the relationship between bacterial (**a**) and fungal (**b**) community structures via T-RFLP in different treatments with three replicates using T-REX software. The explanations of treatment abbreviations are referred to Table 2.

The relationships between microbial community structures in the various treatments were obtained via T-RFLP analysis (Fig. 5). Similar bacterial community structures were observed in the control, RS and nitrogenous amendments at or below 0.50 g N kg^−1^ soil (Fig. 5a). However, with increasing the N level (i.e., 1.00 g N kg^−1^ soil), the bacterial community structures in the CC and AW treatments exhibited substantial variation from the above group. Regarding fungal community structure relationships between the treatments (Fig. 5b), PCA showed a close association among the fugal community structures in the control, RS and all of the nitrogenous amendments at or below 0.25 g N kg^−1^ soil (Fig. 5b). At or above 0.50 g N kg^−1^ soil, the fungal community structure in the A+L and AW treatments showed a close relationship with the above treatments; however, the fungal community in the CC treatments substantially differed from that in the other treatments (Fig. 5b).

**Figure 6:**
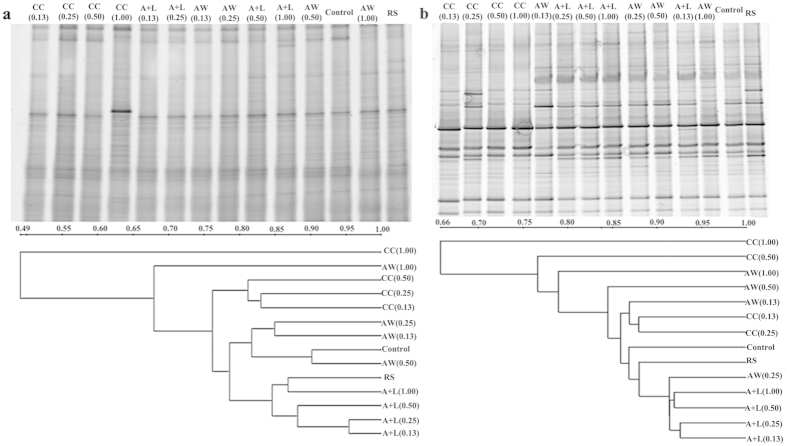
Typical DGGE profiles and the phylogenetic tree of bacterial (**a**) and fungal (**b**) community structures in soil. The explanations of treatment abbreviations are referred to Table 2.

The DGGE profiles of the bacterial and fungal communities in different treatments were also analyzed (Fig. 6). The cluster analysis of bacterial communities showed high similarity between the control, RS and nitrogenous amendments at or below 0.50 g N kg^−1^ soil (dice coefficient >76%) (Fig. 6a). However, when the nitrogen level was increased to 1.00 g N kg^−1^ soil, the similarity reduced in the CC (49%) and AW (68%) treatments but remained high in the A+L treatment (87%) (Fig. 6a). For the fungal community, the community structure was clearly separated according to the treatments (Fig. 6b). Higher dice coefficient values of similarity were observed (dice coefficient >86%) in the control, RS and treatments at or below 0.25 g N kg^−1^ soil. When the nitrogen level was increased to 0.50 g N kg^−1^ soil, the similarity value was reduced in the CC treatment, whereas it showed no substantial variation between the AW, A+L and the control or RS treatments. At the N level of 1.00 g kg^−1^ soil, the dice coefficients of similarity further decreased in the CC and AW treatments but changed very little in the A+L4 treatments.

The diversity indexes of the bacterial and fungal communities were analyzed using the T-RFLP data (Table 1). In the CC and AW treatments at or above 0.50 g N kg^−1^ soil, the richness and Shannon-weaver index of the bacterial community were significantly higher than those in the control and RS treatments, whereas neither of the above indexes in the A+L treatments nor the treatments at or below 0.25 g N kg^−1^ level differed from those in the control or RS treatments. For fungal diversity, nitrogenous amendments at or below 0.25 g N kg^−1^ soil did not change the richness or Shannon-Weaver index. As N levels increased (>0.50 g kg^−1^ soil), the richness and Shannon-Weaver index significantly increased after CC applications but were not affected by A+L or AW applications.

## Discussion

Our study demonstrated that calcium cyanamide had the highest ability, followed by ammonia water, to suppress the survival of *R. solanacearum*. The significantly higher concentrations of soil 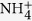 and NO_2_^−^ after their application were two possible explanations for why calcium cyanamide (CC) and ammonia water (AW) were superior to control *R. solanacearum*. Ammonium can reduce the growth of *R. solanacearum* through ammonium toxicity against the pathogen^15^, whereas the nitrite (NO_2_^−^) is toxic to some pathogens by destroying important compounds in organisms^22^,^23^,^24^. The significantly higher pH value in the CC-treated soil may be another reason for the higher efficiency of calcium cyanamide to more strongly inhibit *R. solanacearum* survival than the other two amendments. Michel and Mew^15^ indicated that the initial decrease in the *R. solanacearum* population in MMSU soil collected at Batac (Ilocos Norte Province) was likely due to the high pH. High pH favors the formation of ammonia from ammonium, whereas ammonia is believed to disrupt membrane integrity and eliminate proton gradients across cell membranes^25^,^26^,^27^. Therefore, we speculate that the high soil pH further enhances the ability of calcium cyanamide to control *R. solanacearum*.

In light of the control efficiencies of *R. solanacearum* by the three amendments, CC may be the optimal fumigant for controlling bacterial wilt. However, the cost of the amendments should also be considered. In China, the cost of using CC is approximately three- or four-fold higher than A+L or AW. Our present study suggests that AW application at a rate of 0.25 g N kg^−1^ soil is most suitable for farmers to control bacterial wilt because this treatment has the lowest cost among the treatments that can efficiently suppress the survival of *R. solanacearum*.

In our study, soil microbial activity and community populations were decreased by the application of nitrogenous amendments during the initial period, which was likely due to the formation of ammonium, ammonia, nitrate and nitrite^14^,^18^,^28^; however, they were restored after the initial decrease when the application rate was at or below 0.26 g N kg^−1^ soil. The significant increase in dehydrogenase activity and microbial community populations after CC application above 0.50 g N kg^−1^ soil (Figs 3 and 4) were likely results of the increased pH of the CC-treated soil because pH increase can potentially alter the soil nutrient availability which may benefit other microorganisms but not *R. solanacearum*^15^,^29^,^30^. An increase in microbial activity and community populations generally enhances the competitiveness of indigenous microorganisms against pathogens^31^. Nevertheless, these results indicate that the three nitrogenous amendments were not toxic to microorganisms, with the exception of the suppressive capacity against *R. solanacearum* at or below 1.00 g N kg^−1^ soil. In a certain sense, the effective application rate at a higher N level has the potential capacity to increase suppression against *R. solanacearum*, likely by increasing microbial activity and community populations.

The above results obtained using the traditional plating technique are supported by results from a cluster analysis for DGGE and a principal component analysis for T-RFLP (Figs 5 and 6; Table 1). The three nitrogenous amendments at or below 0.25 g N kg^−1^ soil had little effect on the microbial community. Nevertheless, with increasing CC and AW levels, the significant increases in microbial community diversity indicate that new microorganisms may be involved in the nutrient metabolism of or resistance against *R. solanacearum*. Other studies have shown that an increase in microbial diversity enhanced the soil suppressive capacity against *R. solanacearum*^32^,^33^. Further sequencing of 16S rDNA segments can provide valuable information regarding the effects of nitrogenous amendments on microbial community structure and is warranted in future research. These results further demonstrated that the three nitrogenous amendments at the recommended level suppressed pathogens but had little detrimental impact on microbial community structure after a given incubation period.

Our previous study revealed that biocontrol (bioorganic fertilizer) exhibited a better suppressive efficiency against *R. solanacearum* compared with the organic amendments and local conventional methods^32^. However, the suppressive capacity of biocontrol would be compromised with varying temperature and rainfall conditions^32^,^34^. Yilmaz *et al.*^35^ reported that soil-borne pathogens could not be effectively suppressed with a single management strategy. This study indicated that the nitrogenous amendments could represent an alternative strategy for suppressing the survival of *R. solanacearum*. Therefore, we predict that the combination of nitrogenous amendments and biocontrol can result in enhanced suppression.

In conclusion, the application of calcium cyanamide, ammonia water, or ammonium bicarbonate can effectively suppress *R. solanacearum* at rates of N over 0.13, 0.25, and 0.50 g N kg^−1^ soil, respectively. Calcium cyanamide had the highest ability to inhibit *R. solanacearum* survival, whereas ammonia water was the most economical amendment. Additionally, these two nitrogenous amendments could improve soil N supply and had little detrimental impact on the soil microbial community. Our present study provides basic information for growers when selecting a suitable alkaline nitrogenous amendment and regarding its application rate in controlling bacterial wilt. However, further research on the effectiveness of these nitrogenous amendments in controlling *R. solanacearum* in other soil types and under field conditions is warranted.

## Material and Methods

### Isolation of bacterial wilt pathogen

Bacterial wilt pathogens were isolated from the rhizosphere soil of 50-d-old tomato plants grown in the field in Ningbo, Zhejiang province, China (121°44′ E, 29°56′ N) in which tomato bacterial wilt had become a serious problem. Strains of tomato bacterial wilt were isolated using the semi-selective medium (SMSA)^36^. The isolated strain was recognized by colony morphology characteristics of *R. solanacearum* and by visualizing the approximately 280-bp *R. solanacearum*-specific fragments using the primer pair AU 759/760 under UV light^37^ (Fig. S1a–c). The strain was determined to have the capacity to induce wilt in up to 99% of tomato plants (Fig. S1d). The 16S rRNA gene of this strain was sequenced and was closely related (99.9% similarity) to *R. solanacearum* strain QL-Rs 1115 through a comparison against all sequences in the NCBI database^6^. Therefore, this strain was confirmed to be the pathogen of tomato bacterial wilt and was code-named NB-Rs 1021.

### Incubation experiment

The experimental soil was collected from the top 20 cm of a field plot in Ningbo, Zhejiang province, China, and the soil was covered with plastic for solarization during the summer of 2013. There was no *R. solanacearum* detected in the soil using the plate culture method^36^ and no history of fumigation or the use of calcium cyanamide (CaCN_2_), ammonium hydroxide, lime or ammonia water in the test soil. The soil is a plinthosol (FAO/Unesco) with a pH of 5.45 (1:5 water), an electrical conductivity (EC) of 841 μS cm^−1^ (1:5 water), a total organic C content of 31.4 mg g^−1^ (Walkley-Black method)^38^, a total N content of 1.51 g kg^−1^ (Kjeldahl analysis method)^39^, an available phosphorus content of 512 mg kg^−1^ (extracted via 0.3 M NH_4_F-0.025 M HCl)^40^, and available potassium of 421 mg kg^−1^ (extracted in 1 M NH_4_OAc)^41^.

The 400 g of air-dried soil (<2 mm) was placed in each 500-mL sterile plastic beaker and incubated at 25 °C after adjusting the moisture content to 50% water holding capacity. Following two weeks of incubation, the water suspension of the isolated *R. solanacearum* (RS) was inoculated into soil and incubated for three days for a final bacterial concentration of 6.3 log CFU g^−1^ dry soil. The control soil (without *R. solanacearum*) received the same volume of sterile water. Afterward, three different inorganic nitrogenous amendments [calcium cyanamide (CC) (19.5% N, 55% CaO), ammonium bicarbonate (17.7% N) and lime (60% CaO) mixture (A+L), and ammonia water (AW) (0.902 g mL^−1^, 21.8% N)] at N levels of 0.13, 0.25, 0.50 and 1.00 g kg^−1^ soil, respectively, were mixed into the RS-inoculated soil as described in Table 2. All of the plastic beakers were sealed with polyethylene film to prevent water loss but allow air exchange. The beakers were further incubated at 25 °C under 95% relative humidity. This experiment was performed twice, each with four replicates.

### Sampling

The soil samples were taken at 0, 1, 3, 7, 14, 21, 28 and 35 days after the alkaline nitrogenous treatments. For each soil sample, a portion of fresh soil was directly used for measurements of soil chemical properties, microbial community populations, fluorescein diacetate hydrolysis and dehydrogenase activity; another portion of the soil was stored at -80 °C until the extraction of soil DNA for analysis via PCR-DGGE and T-RFLP.

### Analysis of soil chemical properties

Soil water content was determined after oven drying at 105 °C for 24 h. The soil pH was measured using a pH meter in a water suspension (1:5 soil/water) after shaking for 1 h. The soil 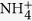-N, NO_3_^−^-N and NO_2_^−^-N were extracted with 2 M KCl and assayed via flow injection analysis^42^.

### Assay of soil fluorescein diacetate hydrolysis and dehydrogenase activity

Fluorescein diacetate hydrolysis was performed using the method described by Adam and Duncan^20^. One gram of fresh soil (<2 mm) were placed in a 50-mL conical flask; 15 mL of 60 mM potassium phosphate buffer (pH 7.6) was then added to the flask. The reaction began after the addition of stock solution (0.2 mL 1,000 mg FDA mL^−1^). The contents of the flask were placed in an orbital incubator (100 rev min^−1^) at 30 °C for 20 min. Then, 15 mL chloroform/methanol (2:1 v/v) was immediately added to the flask to terminate the reaction. The concentrations of fluorescein in the filtrates were measured at 490 nm using a spectrophotometer.

The activity of dehydrogenase in the soil was assayed as described by Tabatabai^43^. Twenty grams of fresh soil was mixed with 0.2 g CaCO_3_, and 6 g of soil was taken from the mixture and placed in a 50-mL conical flask. One milliliter of 0.3% TTC (2,3,5-triphenyltetrazolium chloride) and 2.5 mL distilled water were added to the conical flask. The mixtures were incubated for 24 h at 37 °C. After that, 10 mL methanol was added to the flask to extract the reduced formazan (red color). The red mixture was filtered with cotton in a funnel by continuously adding the methanol. The concentrations of reduced formazan in the filtrates were determined at 485 nm using a spectrophotometer.

### Enumeration of soil microbial community populations

The microbial community populations were enumerated using a standard 10-fold dilution method^4^. Briefly, 10 g of fresh soil were transferred into a 250-mL conical flask containing 90 mL of sterile distilled water. The flasks were shaken on a rotary shaker at 200 rpm for 30 min. Soil suspensions at appropriate dilution rates were spread on plates and incubated in respective media as described below.

### *R. solanacearum* population

Semi-selective medium (SASM) was used for the measurement of *R. solanacearum* populations^36^. After incubation at 30 °C for 2 days, the population of *R. solanacearum* was determined.

### Bacterial, fungal and actinomycetes community populations

Beef extract-peptone medium was used for the bacterial population, Martin’s Rose Bengal agar was used for the fungal population, and Gaoshi No. 1 agar was used for the population of actinomycetes^44^. After incubation at 30 °C for 2 d, the population of bacteria was determined. The inoculated agar plates with fungi were incubated in the dark at 28 °C for 3 d, whereas the plates with actinomycetes were incubated at 30 °C for 5 d.

### Soil microbial community fingerprinting via PCR-DGGE and T-RFLP

The DNA was extracted from 0.5 g soil samples using the UltraClean Soil DNA Isolation Kit (MO BIO Laboratories Inc., Carlsbad, CA, USA) according to the manufacturer’s instructions.

For denaturing gradient gel electrophoresis (DGGE) of the microbial community, polymerase chain reaction (PCR) targeting bacterial 16S rRNA was performed with primers 338f-GC/518r^45^. The first PCR amplification of fungal internal transcribed spacer (ITS) rRNA regions used the primers ITSF^46^ and ITS4^47^. The next PCR amplifications in fungi were performed to produce ITS1 products^32^. The mixture and conditions of PCR amplification for the bacterial and fungal communities followed the description by Liu *et al.*^32^.

Terminal restriction fragment-length polymorphism (T-RFLP) analysis was applied to characterize the bacterial and fungal community structures in different treatments. PCR amplification of the bacterial 16S rRNA gene was performed with forward primer 12f (5′-AGA GTT TGA TCC TGG CTC AG-3′) and reverse primer 1492r (5′-GGT TAC CTT GTT ACG ACT T-3′) under the following cycle conditions: 94 °C for 5 min, then 35 cycles of 94 °C for 30 s, 55 °C for 30 s, and 72 °C for 30 s, followed by a final extension step at 72 °C for 5 min. Primers PN3 (5′-CCG TTG GTG AAC CAG CGG AGG GAT C-3′) and PN34 (5′-TCC GCT TAT TGA TAT GCT TAA G-3′) were used for PCR amplification of the fungal targeting ITS rRNA regions according to the conditions described by Viaud *et al.*^48^. PCR products of bacteria and fungi were purified and then digested separately with tetrameric restriction endonuclease HaeIII at 37 °C for 4 h. Aliquots (8 μL) of the restriction digests were examined via 2% agarose gel electrophoresis stained using SYBR Green I. Phylotype richness was calculated as the total number of distinct TRF sizes in a profile. The Shannon-Weiner diversity index (*H*) was calculated as follows:


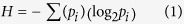


where *p* is the proportion of an individual peak height relative to the sum of all peak heights.

### Statistical analysis

The data analysis was performed with the SPSS 13.0 software program (SPSS Inc., Chicago, IL). ANOVA was used to assess differences in soil biochemical properties at each bioassay. A comparison of means was performed using the Duncan multiple range test with a significance level of *P* < 0.05. A principal component analysis was performed to determine the relationship between microbial community structures derived from T-RFLP in different treatments using T-REX software (http://trex.biohpc.org/). DGGE images were analyzed for band detection and intensity with Quantity One software (Version 4.6.3, Bio-Rad Laboratories). Cluster analysis for DGGE was performed with the UPGMA algorithm incorporating Jaccard’s coefficient of similarity.

## Additional Information

**How to cite this article**: Liu, L. *et al.* Effect of calcium cyanamide, ammonium bicarbonate and lime mixture, and ammonia water on survival of *Ralstonia solanacearum* and microbial community. *Sci. Rep.* **6**, 19037; doi: 10.1038/srep19037 (2016).

## Supplementary Material

Supplementary Information

## Figures and Tables

**Table 1:** Richness and diversity Shannon-Weaver index (*H*) of microbial community under various treatments through the analysis of T-RFLP.

Treatment	Bacteria		Fungi	
	Richness	*H*	Richness	*H*
CC (0.13)	10 ± 2bc	1.49 ± 0.07d	17 ± 2c	3.08 ± 0.08c
CC (0.25)	10 ± 1c	1.51 ± 0.04d	16 ± 2c	3.09 ± 0.07c
CC (0.50)	13 ± 1ab	2.15 ± 0.11b	21 ± 1b	3.39 ± 0.04b
CC (1.00)	15 ± 2a	2.86 ± 0.13a	25 ± 1a	3.65 ± 0.08a
A+L (0.13)	10 ± 2bc	1.47 ± 0.10d	18 ± 1c	3.13 ± 0.05c
A+L (0.25)	9 ± 2c	1.41 ± 0.07c	17 ± 2c	3.09 ± 0.07c
A+L (0.50)	11 ± 1bc	1.57 ± 0.08d	19 ± 2bc	3.16 ± 0.08c
A+L (1.00)	11 ± 1bc	1.50 ± 0.05d	20 ± 1bc	3.21 ± 0.07c
AW (0.13)	9 ± 1c	1.40 ± 0.07d	17 ± 2c	3.16 ± 0.09c
AW (0.25)	9 ± 2c	1.49 ± 0.09d	18 ± 1c	3.13 ± 0.04c
AW (0.50)	13 ± 1ab	1.76 ± 0.03c	18 ± 2bc	3.19 ± 0.06c
AW (1.00)	14 ± 2ab	1.82 ± 0.04c	19 ± 1bc	3.20 ± 0.09c
RS	9 ± 2c	1.44 ± 0.11d	17 ± 2c	3.10 ± 0.09c
Control	10 ± 1c	1.48 ± 0.09cd	16 ± 2c	3.06 ± 0.07c

Data are the means ± SD (n = 4). The data within one column of each trial with the same letters did not significantly differ (Duncan’s significance level of 0.05). The explanations of treatment abbreviations are referred to Table 2.

**Table 2:** The experimental treatments (types and N application rates) of nitrogenous amendments in *R. solanacearum*-inoculated soil.

	Nitrogenous amendment	Application rate	Treatment abbreviation and (N rate in g kg^−1^)
Soil without *R. solanacearum* inoculation	—	—	Control
Soil with *R. solanacearum* inoculation	—	—	RS
	calcium cyanamide (g kg^−1^)	0.65	CC (0.13)
		1.3	CC (0.25)
		2.6	CC (0.50)
		5.2	CC (1.00)
	Ammonium bicarbonate (g kg^−1^) + lime mixture (g kg^−1^)	0.72 + 0.60	A+L (0.13)
		1.4 + 1.2	A+L (0.25)
		2.8 + 2.4	A+L (0.50)
		5.6 + 4.8	A+L (1.00)
	Ammonia water (mL kg^−1^)	0.65	AW (0.13)
	1.3	AW (0.25)	
	2.6	AW (0.50)	
	5.2	AW (1.00)	

— denotes no nitrogenous amendment added to the soil.
