# Supplementary material for: Effect of calcium cyanamide, ammonium bicarbonate and lime mixture, and ammonia water on survival of Ralstonia solanacearum and microbial community
Source: Sci Rep. 2016 Jan 7;6:19037. doi: 10.1038/srep19037 (PMC4704052; doi:10.1038/srep19037)
Supplement: Supplementary Information [file srep19037-s1.pdf]

Supplementary Information

Title:

Effect of calcium cyanamide, ammonium bicarbonate and lime mixture, and ammonia water on survival of *Ralstonia solanacearum* and microbial community

Authors:

Lijuan Liu[1, a], Chengliang Sun[1, a], Xingxing Liu[2], Xiaolin He[1], Miao Liu[1], Hao Wu[1], Caixian Tang[3], Chongwei Jin[1,*] Yongsong Zhang[1,*]

Institutions:

[1] Ministry of Education Key Laboratory of Environmental Remediation and Ecosystem Health, College of Environmental and Resource Sciences, Zhejiang University, Hangzhou 310058, China;

[2] Zhejiang Provincial Key Laboratory of Subtropical Soil and Plant Nutrition, College of Environmental and Resource Sciences, Zhejiang University, Hangzhou 310058, China;

[3] Centre for AgriBioscience/Department of Agricultural Sciences, La Trobe University, Melbourne Campus, Bundoora, Vic 3086, Australia

[a] These authors contributed equally to this work.

[*] To whom correspondence should be addressed

Chongwei Jin, E-mail: jincw@zju.edu.cn; Yongsong Zhang, yszhang@zju.edu.cn

Supplementary Fig. S1

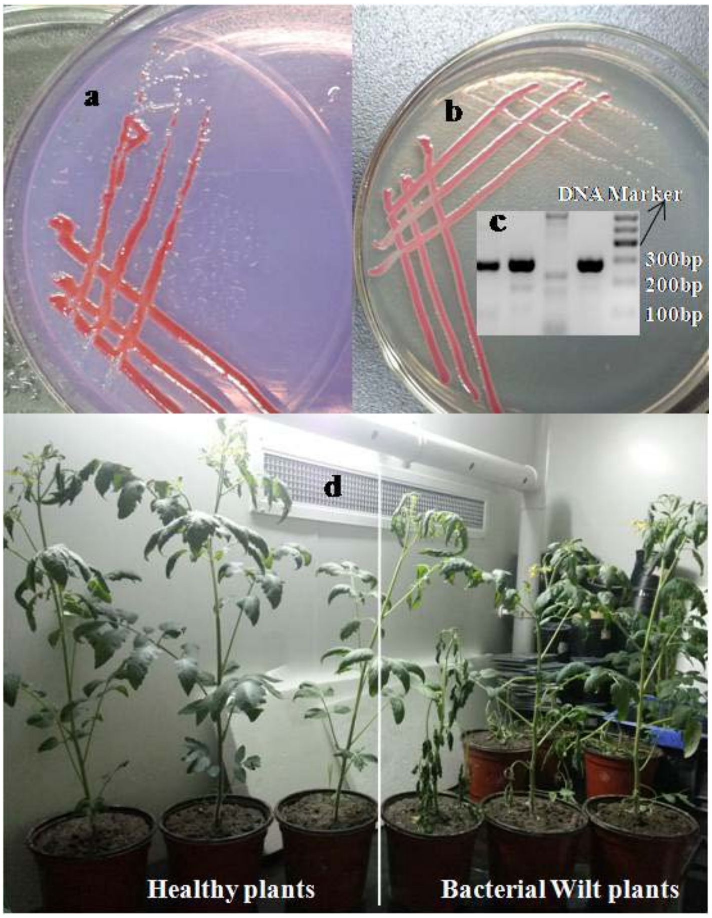

Fig. S1 *R. solanacearum* colony morphology (a and b), agarose electrophoresis image of *R. solanacearum*-specific fragments using the primer pair AU 759/760 (c) and bacterial wilt symptoms of 30-d-old tomato plants (d).
